# Regenerative Medicine, Stem Cell Therapies, and Platelet-Rich Plasma: Where Is the Evidence?

**DOI:** 10.1093/asj/sjz317

**Published:** 2020-01-13

**Authors:** Naveen Virin Goddard, Norman Waterhouse

**Affiliations:** 1 Undergraduate Medical Student, Birmingham Medical School, College of Medical and Dental Sciences, University of Birmingham, Edgbaston, Birmingham, United Kingdom; 2 Consultant Plastic Surgeon, Wellington Hospital, London, United Kingdom

We live in the age of unreason where, as Martha Gill observes, homeopaths and anti-vaxxers are put on an equal footing with groundbreaking research.^1^ There is little consensus or consistency of thought based on science. France has stopped funding homeopathy, yet in the United Kingdom, homeopathy has royal patronage.^2^ On the other hand, one-third of French people believe that vaccines are dangerous.^3^ As Carrie Dennett, a leading dietician nutritionist has noted, “the more you hear information repeated, even if its misinformation, the more likely you are to believe it.” ^4^ Familiarity breeds acceptance. Health information peddled by celebrities has more traction than if proposed by medical authorities. This results in claims of the benefits of vaginal steaming and jade eggs, which remain to be verified.^5^

It is undeniable that in the current aesthetic arena, many treatment modalities are carried out despite the absence of peer-reviewed evidence or clinical reviews to support their efficacy. Many therapies are widely advertised and advocated on social media, and after a period of time the therapy becomes accepted without any preexisting evidence of direct benefit or any data regarding complications.

The power of social media is seen perhaps most obviously with the anti-vaccination lobby, who continue to claim that vaccination is the cause of many problems, including autism, despite the discreditation of the original research that suggested this was the case.^6,7^

Ignoring the weight of peer-reviewed evidence in this case has led to the detriment of children’s health as noted by an increase in the incidence of measles in the pediatric population.^8^ If, as medical professionals—and for the purpose of this article, plastic and aesthetic surgeons—we are to hold any credibility regarding the efficacy of emerging and indeed established therapies, we need to be clear that we will not endorse them because they are lucrative and neither will we “partner” with celebrities to promote them.

Regrettably, the specialty of plastic surgery and aesthetic surgery has a poor history regarding the introduction of therapies on the basis of little evidence. Historically this dates back as far as the initiation of breast augmentation.^9^ In a specialty where it can be very difficult to determine outcome measures successfully, many treatments have gained a foothold in the armamentarium on the back of minimal, perfunctory research.

Commercial pressures often take precedence over the normal processes of awaiting results of peer review outcome studies. In many situations, such studies are not available at all. Many therapeutic nonsurgical gestures and modalities are brought straight to market, bypassing any form of regulation or evidence and have been free to offer to patients. The obvious example is nonsurgical fillers. Their designation as a medical device rather than a drug allowed their passage to market unencumbered by any meaningful clinical evaluation. Permanent and semipermanent fillers have been responsible for significant morbidity and adverse outcomes as the behavior of the material became apparent over time.^10-12^ The lack of research and also the lack of uniformity, even as to the modality of administration, often makes unpicking the causality for complications difficult.

Aesthetic medicine is commercially driven by the “latest” treatments, often marketed with a brand name and celebrity endorsement via social media. Once they become recognized in the marketplace, requests for treatment are predominantly patient driven. Clinicians may adopt the therapy for fear of losing market share or not being seen to operate at the cutting edge of new developments. The aggressive marketing of thread lifting is a perfect example.

Currently, there is a new modality in town: stem cell therapy and regenerative medicine. Regenerative medicine and regenerative therapies are new terms and indeed new specialties that have exploded in terms of laboratory research and funding. The route to the market has been swift, with therapies on sale for several clinical applications.^13^ The enthusiasm for the potential of cell-based therapies is frequently directed at clinical need, where at present available medical and pharmacological solutions are unsatisfactory or imperfect. Targeted patient groups are often those with chronic diseases such as rheumatoid arthritis.^14^

The haste to market a clinical application in a wide variety of medical conditions drives the enormous number of cellular therapies in the process of being developed. This enthusiasm should be tempered by medical professionals who understand that rigid research and data should be available before the therapies are introduced. Robert Alexander in the paper “Biocellular Regenerative Medicine: Use of Adipose-Derived Stem/Stromal Cells and It’s Native Bioactive Matrix” states that when discussing the value of adipose stromal cell vascular fraction, there is “an information gap between clinical applications and those strictly of research value. If clinicians read only the peer-reviewed clinical journals, they miss up to 85% of the pertinent information and data evolving on almost a daily basis, because the important advances appear in basic scientific and engineering publications.” ^15^

How antithetical and audacious is the suggestion that if only physicians would ignore data in peer review clinical trials and simply take the word of research papers that appear in engineering and basic science journals, they would be more inclined to immediately apply these findings to the clinical arena? The author further suggests that it is the clinicians themselves who are causing problems for their patients by waiting for peer review clinical data. If this view gains traction, we risk abandoning the process by which we evaluate any treatment for clinical use.

The consequences of clinical enthusiasm over tried and tested evaluation have been dramatically demonstrated by the events surrounding Paolo Macchiarini and the Karolina Institute. Macchiarini, a Swiss-born Italian thoracic surgeon, headed a research laboratory in regenerative medicine. He persuaded colleagues that he could reconstruct the trachea utilizing a synthetic scaffold seeded with stem cells, which would become incorporated into the patient’s own tissue and survive and function as respiratory epithelium. This proposition was put into clinical practice, but the outcomes were not as expected and the anticipated results were tragically optimistic. Seven of the 9 patients who underwent the procedure died.^16,17^ Subsequently, Macchiarini has been found guilty of falsifying his curriculum vitae, and scientific misconduct by Macchiarini and his co-authors has been discovered in 6 papers reviewed by Sweden’s National Scientific Review Board. They have since been retracted.^18^

In aesthetic medicine there are less potentially fatal consequences of utilizing “stem cells” in the aesthetic arena, which makes it more difficult to temper the enthusiasm of clinicians. The potential benefits of regenerative medicine appear to be lucrative, and many respected institutions including the Mayo Clinic, Cleveland Clinic, and University of Miami have entered the field of stem cell research and related therapies.

In the United States, these treatments are not recognized for remuneration by insurance companies and are therefore self-paid by patients. This provides an extra source of revenue for clinicians and institutions from a vulnerable group of patients. Education for doctors on the stem cell market remains scarce. In chronic conditions such as arthritis, where the treatment options may be limited, many institutions have utilized marketing videos interviewing doctors and patients with apocryphal stories of great clinical benefit.^19^ These videos are “infomercials” and have attracted the attention of federal regulators in the United States concerned about their accuracy.^20,21^ Critics have suggested that hospitals may be exploiting vulnerable patients and profiting from trendy but unproven treatments. The Food and Drug Administration is currently attempting to shut down clinics that hawk unapproved stem cell therapies.^22^

Although there may be a comfort for aesthetic doctors that the potential downsides and morbidities are few, it should be recognized that there have been 3 cases of blindness and multiple other cases of serious infections and neoplasia resulting from stem cell treatments.^23^ Two patients in New Mexico were recently diagnosed with HIV infections following platelet-rich plasma (PRP) treatments for facial rejuvenation.^24^

A review paper by Leigh Turner, Associate Professor at the University of Minnesota, has documented the dynamics and size of the stem cell market and observes that there is no evidence that they work.^25^ Many stem cell clinics state that they do not need FDA approval, because the cells are harvested and returned within the “same surgical procedure,” thus negating the need for a biological license. Although the FDA states that it disagrees with this position, at the time of writing, only a handful of clinics have received warnings.

There is now a growing sentiment among physicians that the FDA must be more proactive in its oversight of clinics offering stem cell products and therapies.^26^ This could be achieved by enlisting the help of national surgical facility-accrediting agencies, state legislatures, and medical boards to facilitate the registration and licensing of facilities with a recognized national organization.

There is, of course, a real prospect that the biotech industry may in the future develop valid and alternative means of dealing with chronic conditions, which, at the present time, are not well served by medicine. FDA approval has been granted for a few therapies, including T-cell-based immunotherapies for certain malignancies, for skin regeneration after burn injuries, and autologous scaffold for knee cartilage. Approved uses are listed on the FDA website.^27^

In California, the Institute of Regenerative Medicine (created in 2004) has been funded with $3.4 billion public dollars.^28^ The industry has its own sector of companies focused on research, development, and commercialization of regenerative cellular therapies that they believe will transform the practice of medicine. At present, the commercial aspects of regenerative medicine are fueling hype and misinformation, and there are many attempts to sell invalid and unapproved stem cell and regenerative therapies.

In the field of cosmetic surgery, attention has been largely directed to the benefits of injecting adipose tissue in a variety of clinical situations and also the utilization of concentrated platelets, so-called PRP therapy.^29^ In the speciality of plastic surgery, the most topical therapeutic application of an unproven treatment is the utilization of PRP.

PRP has rapidly assumed a position of respectability and is increasingly utilized by mainstream clinical practitioners for a wide variety of conditions. However, the evidence for its efficacy remains marginal and, in the vast majority of cases, inadequate.^30-32^ Simply by being present in the market for a certain amount of time and by being widely disseminated on social media, PRP appears to have credibility as a therapy that its research history does not justify.

Much of the research around PRP has concentrated on its effect in conjunction with fat transfer, particularly when comparing the outcome of fat transfer against fat transfer with PRP therapy to determine whether the PRP enhances fat survival.^33^ This arena is itself problematic, because there is no well-recognized and accepted protocol for injecting fat.^34^ It can be centrifuged or noncentrifuged, strained with antibiotics, multiply strained to micro-fat or nano-fat through cannulae of increasingly narrow diameter, injected as a single pass or multiple pass, and with many other variants. There is equally no acceptable figure for fat transfer success rates.^35^ It is therefore difficult to ascribe improvement in fat grafting success rates to the presence or absence of PRP when multiple modalities of treatment with no universally accepted parameters of outcome exist.

PRP therapy is now utilized for several aesthetic treatments marketed for soft tissue rejuvenation. The therapeutic gesture is delivered by injecting a concentrate of platelets extracted from plasma into a target area.^36^ Once activated, the large number of growth factors and cytokines released by the platelet alpha-granules are said to have beneficial effects in soft tissue healing. These principles have been applied in a number of different clinical environments.^37^

It is conceded that some studies do seem to show a beneficial effect from the injection of PRP in the treatment of androgenic alopecia, although it is difficult to understand why this might be true.^38,39^ It is interesting to note that PRP therapies have found application in conditions in vulnerable patient groups, such as female sexual arousal disorder. The research related to its utilization in these patients is, however, inadequate to the point of nonexistent. The “paper” documenting its benefits would meet no peer review criteria and yet has been utilized as a justification to inject PRP with hyaluronic acid into the genitalia of women supposedly suffering from this disorder.^40,41^ It has also been applied and injected into the root of the penis to enhance sexual function in men.^42^

There is an apocryphal belief that PRP has been employed to heal the injured tendons of expensive racehorses. Although studies in the veterinary literature demonstrating some favorable outcomes do exist, these studies are overwhelmingly of poor quality and PRP is not a mainstream treatment for tendon injuries in veterinary medicine.

The first published utilization in humans was reported nearly 30 years ago in the field of cardiothoracic surgery.^43^ However, PRP is being employed increasingly in the specialty of orthopedics for chronic inflammatory and degenerative conditions. In this specialty, many clinicians have carried out peer review studies in a responsible and scientific manner, and it is interesting that these papers almost universally demonstrate that PRP has no value compared with a placebo.^32,44^ There are few if any chronic conditions in orthopedics for which PRP has been accepted as a useful therapy.^45^

PRP has become popular as a facial injection of concentrate into the face to rejuvenate the soft tissue and the skin. This technique, given a commercial brand name of a Vampire Facial, has attracted celebrity endorsements by the Kardashian family. However, Kim Kardashian subsequently disclaimed The Vampire Facial, claiming that “it’s the one treatment that I’ll never do again’.^46,47^

Studies purporting to show clinical benefits with PRP in facial rejuvenation often lack controls as well as objective assessment before and after the intervention and describe inconsistent methods for preparation and application.^48,49^ Complication rates are rarely discussed or published, although there are many reports of severe inflammatory and allergic reactions in patients who underwent PRP injections, and PRP treatment has been implicated in the development of HIV infection in 2 patients attending a Vampire Facial clinic in New Mexico.^50-52^ Overall, the utilization of PRP is indiscriminate and unregulated. The function and mechanism of the action of platelets in this regard is poorly understood, and as an adjunct to fat transfer the manner of preparation is variable.^53-56^

Each commercial separation system designed to produce PRP yields plasma of varying cellular composition.^57^ Furthermore, although there is some evidence suggesting that concomitant use of nonsteroidal antiinflammatory drugs does not affect the serum level of growth factors in PRP, more evidence is needed to outline the possible effects of other commonly prescribed medications that influence platelet aggregation and the coagulation cascade.^58-60^

The vast majority of so-called scientific publications on the benefit of PRP demonstrate poor statistics, observer bias, and flawed protocols.^61^ The evidence that elucidates its mechanism and preparation remains vague and lacks uniformity. PRP is currently one of the more popular products that has been brought to market on the basis of falsified or propagandized information with almost zero clinical evidence.

There may be a future for PRP, but it is far from proven. As responsible professionals, we should step out of the queue to buy PRP machines until the clinical benefits are properly clinically evaluated and published. Otherwise, we risk abdicating our position as guardians of patient safety and have no more authority than Gwyneth Paltrow or Prince Charles.

## Disclosures

The authors declared no potential conflicts of interest with respect to the research, authorship, and publication of this article.

## Funding

The authors received no financial support for the research, authorship, and publication of this article.
